# Everything Comes with a Price: The Toxicity Profile of DNA-Damage Response Targeting Agents

**DOI:** 10.3390/cancers14040953

**Published:** 2022-02-14

**Authors:** Federica Martorana, Leandro Apolinario Da Silva, Cristiana Sessa, Ilaria Colombo

**Affiliations:** 1 Department of Clinical and Experimental Medicine, University of Catania, 95123 Catania, Italy; fede.marto.fm@gmail.com; 2 Service of Medical Oncology, Oncology Institute of Southern Switzerland (IOSI), EOC, 6500 Bellinzona, Switzerland; leandroapolinario83@hotmail.com (L.A.D.S.); cristiana.sessa@eoc.ch (C.S.)

**Keywords:** DNA damage response inhibitors, PARP inhibitors, ATR inhibitors, CHK1 inhibitors, WEE1 inhibitors, adverse events, safety profile

## Abstract

**Simple Summary:**

DNA damage induces genome instability, which may elicit cancer development. Defects in the DNA repair machinery further enhance cancer predisposition, but can also be exploited as a therapeutic target. Indeed, targeted agents against specific components of DNA repair, such as PARP inhibitors, are employed in various tumor types, while others, such as ATR, CHK1 or WEE1 inhibitors, are in clinical development. Even though these molecules have proven to be effective in different settings, they display several on- and off-target toxicities, shared by the whole pharmacological class or are drug specific. Among these effects, hematological and gastrointestinal toxicities are the most common, while others are less frequent but potentially life-threatening (e.g., myelodysplastic syndromes). Particular caution is needed in the case of combinatorial therapeutic approaches, which are currently being developed in clinical trials. In any case, it is necessary to recognize and properly manage adverse events of these drugs. This review provides a comprehensive overview on the safety profile of DDR-targeting agents, including indications for their management in clinical practice.

**Abstract:**

Targeting the inherent vulnerability of cancer cells with an impaired DNA Damage Repair (DDR) machinery, Poly-ADP-Ribose-Polymerase (PARP) inhibitors have yielded significant results in several tumor types, eventually entering clinical practice for the treatment of ovarian, breast, pancreatic and prostate cancer. More recently, inhibitors of other key components of DNA repair, such as ATR, CHK1 and WEE1, have been developed and are currently under investigation in clinical trials. The inhibition of DDR inevitably induces on-target and off-target adverse events. Hematological and gastrointestinal toxicities as well as fatigue are common with all DDR-targeting agents, while other adverse events are drug specific, such as hypertension with niraparib and transaminase elevation with rucaparib. Cases of pneumonitis and secondary hematological malignancies have been reported with PARP inhibitors and, despite being overly rare, they deserve particular attention due to their severity. Safety also represents a crucial issue for the development of combination regimens incorporating DDR-targeting agents with other treatments, such as chemotherapy, anti-angiogenics or immunotherapy. As such, overlapping and cumulative toxicities should be considered, especially when more than two classes of drugs are combined. Here, we review the safety profile of DDR-targeting agents when used as single agents or in combination and we provide principles of toxicity management.

## 1. Introduction

DNA replication is an error-prone process, which requires a highly proficient system to recognize and correct these errors to maintain genome stability. Hence, DNA-damage repair (DDR) represents a complex machinery with a crucial role for cell survival [1]. Additionally, DDR is tightly connected with other biological pathways, such as cell cycle, immune system and apoptosis [1]. These interconnected processes are coordinated by several proteins with different roles [2]. Defects in the DDR machinery are common in cancer cells, leading to the accumulation of molecular alterations, which eventually sustain carcinogenesis and tumor progression [3]. At the same time, an impaired DDR process increases cancer cell vulnerability towards cytotoxic compounds, such as platinum-based chemotherapy [3]. Similarly, DDR-targeting agents are highly effective in tumors with alterations in the DNA repair machinery, such as those with *Breast Cancer* (*BRCA*) *1* or *2* genes mutations (*BRCA1/2*). This mechanism of action is called “synthetic lethality” and consists of targeting a biological process, which is vital for cancer cells with pre-existing defects, while sparing the other cells [3,4].

**Figure 1:**
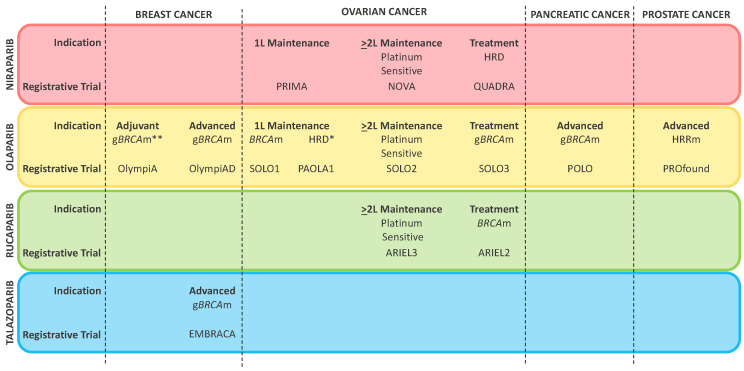
Currently approved indications of PARP inhibitors according to the Food and Drug Administration. * Olaparib in combination with bevacizumab; ** This indication has not been approved yet, but has been granted an accelerated review process by the Food and Drug Administration on 30 November 2021. Figure Legend: 1L first line therapy; 2L second line therapy; BRCAm: BRCA mutated (germline or somatic); gBRCAm: germline BRCA mutated; HRD homologous recombination deficiency; HRRm: homologous recombination repair mutated.

Poly-ADP-Ribose-Polymerase (PARP) inhibitors represent the first class of molecules developed to exploit synthetic lethality in solid tumors [5]. Besides inhibiting PARP enzymes involved in single strand breaks (SSBs) identification and base excision repair (BER), these compounds are responsible for “PARP-trapping”, which implies the formation of highly cytotoxic complexes at the sites of DNA damage [6]. Poly-ADP-Ribose-Polymerase inhibitors have shown substantial efficacy in several tumor types and some have received regulatory approval for the treatment of ovarian, breast, pancreatic and prostate cancer (Figure 1) [7,8,9,10,11,12,13,14,15,16,17,18,19].

More recently, other proteins involved in DDR, such as Ataxia Telangiectasia and Rad3-Related Protein (ATR), Checkpoint Kinase 1 (CHK1) and WEE1 G2 Checkpoint Kinase (WEE1), have been exploited as therapeutic targets [7]. These kinases are implicated in DDR but also in sensing replicative stress and in regulating cell-cycle progression through specific checkpoints [1,20]. Inhibitors of ATR, CHK1 and WEE1 showed preliminary signs of activity in early phase trials, but none of them have entered clinical practice yet [7,21,22,23,24].

As expected, the inhibition of the DNA repair machinery causes different adverse events (AEs), both on- and off-target, class-related or drug-specific events [25]. Promptly recognizing and properly managing these AEs is pivotal to guarantee an adequate treatment exposure. Here, we review the safety profile of DDR-targeting agents, presenting the pathogenesis, incidence and management of the main toxicities reported in clinical trials. We also discuss AEs of DDR-targeting agents in combination with chemotherapy, anti-angiogenics, immunotherapy and other drugs. Lastly, we briefly report about potential biomarkers for the identification of subjects at increased risk of toxicity, which may sustain treatment personalization.

## 2. Safety Profile of DDR-Targeting Agents

### 2.1. Frequent Adverse Events

Currently, a consolidated body of evidence exists about PARP inhibitors AEs, along with specific indications for their prevention, monitoring and management [25,26]. However, few data are available about the safety profile of other DDR-targeting agents, such as ATR, CHK1 or WEE1 inhibitors. Here, we summarize the main toxicities of these compounds, including those commonly occurring (e.g., hematological and gastrointestinal disorders, fatigue) and those rarer but particularly relevant, such as pneumonitis or secondary malignancies.

#### 2.1.1. Hematologic Toxicities

Hematological AEs, including anemia, thrombocytopenia and neutropenia, are common among patients receiving DDR inhibitors and represent on-target class effects of these drugs (Table 1). Pre-clinical studies demonstrated that the direct inhibition of PARP, as well its trapping, are responsible for the hematological toxicities of PARP inhibitors [27,28,29]. Similarly, CHK1 is involved in the maintenance of normal hematopoiesis and this may explain the toxic effects of its inhibition in blood cells [30,31]. Among patients receiving PARP inhibitors, hematological toxicities typically present during the first months of treatment and tend to improve over time, while the kinetic of these toxicities with the other DDR-targeting agents needs further evaluation [25,26].

Anemia. In randomized phase III trials of PARP inhibitors, anemia represented the most frequent hematological toxicity [32]. Any-grade hemoglobin level decreases occurred in about 40% of the enrolled patients, with the lowest rate reported with veliparib in the VELIA trial (17%) and the highest with niraparib in the PRIMA study (63%) [14,33]. Consistently, grade (G) ≥ 3 anemia according to Common Terminology Criteria for Adverse Events (CTCAE), which presented in about 20% of subjects, was less frequent with veliparib (7% in the VELIA trial) and more common with niraparib (31% and 25% in the PRIMA and NOVA trial, respectively) [14,33,34]. Decrease of hemoglobin level was also common in early phase trials evaluating the ATR inhibitors elimusertib and bezosertib [35,36], CHK1 inhibitor prexasertib [23,37,38,39] and WEE1 inhibitor adavosertib [40,41,42]. However—with the exception of elimusertib, which determined an 81% rate of G3/4 anemia—this AE was mainly low grade [23,35,36,37,38,39,40,41,42]. Anemia should be managed with dose interruption according to the hemoglobin levels. Upon recovery, treatment can be resumed at the same dose level at the first occurrence or at a lower dose for recurrent events. Transfusion support should be considered for hemoglobin levels of ≤7 g/dL or higher in patients with symptoms and pre-existing comorbidities, such as cardiac or lung diseases. Erythropoiesis-stimulating agents are not routinely recommended in this setting, while iron, folate and vitamin B12 deficiency should be ruled out and corrected when needed [26,43].

Thrombocytopenia. Thrombocytopenia represents another common toxicity of DDR-targeting agents. In phase III randomized trials, 46−73% of patients treated with niraparib presented any-grade platelet count decreases, with G ≥ 3 events peaking 42% in the PRIMA study [14,44,45]. Subjects with baseline platelet counts <150,000/mm^3^ and body weight < 77 Kg present an increased risk of thrombocytopenia and they should start niraparib at the dose of 200 mg instead of 300 mg [51]. In the EMBRACA trial, 27% of patients treated with talazoparib experienced any-grade platelet count decreases, with 11% at G3 and 4% at G4 [19]. Thrombocytopenia was less frequent in phase III randomized trials of other PARP inhibitors, with a G ≥ 3 rate below 10% (Table 1). Decreased platelet counts were also observed in early phase studies of elimusertib [35], prexasertib [23,37,38,39] and adavosertib, where it was mainly G1-2 [40,41,42,52]. In case of thrombocytopenia, treatment should be withheld for values <100,000 with niraparib and <50,000 with olaparib, rucaparib and talazoparib, and then restarted at the same or at a lower dose level after count normalization [25,26]. No specific indications currently exist for the management of thrombocytopenia induced by other DDR-targeting agents. However, transfusion should be considered in any case of platelet counts <10,000. Higher threshold for transfusion is appropriate in case of active bleeding or fever, while patients on anti-coagulants or anti-platelet medications should discontinue them [26].

Neutropenia. Neutrophil count decreases were also observed in randomized phase III trials of PARP inhibitors, especially with niraparib and talazoparib, but G ≥ 3 events were not frequent and febrile neutropenia was rare [25,26]. Similar results emerged in early phase trials with the WEE1 inhibitor adavosertib [40,41,42,52], while neutropenia was common and often severe with elimusertib and prexasertib [23,35,37,38,39]. Indeed, the rate of G3/4 events was 54% with the ATR inhibitor and reached 93% with the CHK1 inhibitor. However, febrile neutropenia was uncommon, even in this case [23,35,37,38,39]. Neutrophil count decrease should be managed with dose interruption and eventually reduction [26]. Prophylaxis with granulocyte colony-stimulating factor is not routinely recommended for PARP inhibitors [26], even though it was frequently used in prexasertib trials due to the high rate of G4 neutropenia [23,35,37,38].

#### 2.1.2. Gastrointestinal Toxicities

Nausea and vomiting are common gastrointestinal (GI) toxicities induced by PARP inhibitors. They tend to occur early and progressively improve but can be persistent over time [53,54,55]. Other AEs, such as diarrhea, constipation, dyspepsia, dysgeusia and decreased appetite, have been variably reported with different PARP inhibitors [25,53,54]. Gastrointestinal disorders have also been observed in early phase studies with other DDR-targeting agents [23,35,37,38,39,40,42,52].

Emesis. In phase III randomized trials, nausea was present in about 60% of patients treated with PARP inhibitors (43–78%), while vomiting occurred in 20% to 40% of them. However, G3/4 events were infrequent, with an incidence <5% in all trials (Table 1). Given these data, PARP inhibitors are considered moderately emetogenic, with the exception of talazoparib, which displays a lower risk of nausea and vomiting [19,26,56]. Similar incidence of emesis was observed with elimusertib [35] and adavosertib [41,42,52], while it was less frequent with prexasertib, which rarely determined vomiting and G3/4 events [23,37,38,39]. General recommendations to prevent PARP inhibitor-induced emesis include taking the drug after a light meal, eating small portions of food and maintaining adequate hydration [26,43,55]. Even though guidelines suggest daily prophylaxis with 5-hydroxitryptamine 3 receptor (5-HT3) antagonists (ondansetron, granisetron) for oral compounds of moderate emetogenic potential, this is not a standard practice with PARP inhibitors [26,43,56]. In case of G1 toxicity, treatment should be continued, adding anti-emetic medications (e.g., prokinetics, corticosteroids, benzodiazepines) if needed. Neurokinin inhibitors, aprepitant and netupitant, should not be administered concomitantly with olaparib or rucaparib, because of pharmacological interactions [26,55]. Dose interruption, and eventually dose reductions, should be considered for G2 toxicities, especially if supportive measures are ineffective. Treatment must be withheld in case of G ≥ 3 emesis, and it should be interrupted if nausea and vomiting do not improve after 28 days of discontinuation [25,26,43,55].

Diarrhea. About 25% of patients receiving a PARP inhibitor in a randomized phase III trial experienced any-grade diarrhea. Incidence was higher with olaparib and rucaparib, but G ≥ 3 events were uncommon (0–3%) (Table 1) [8,57,58]. This AE was much more frequent with other DDR-targeting agents, especially with adavosertib (up to 85% of any-grade diarrhea, G3/4 7%) [41,42,52], but also with elimusertib and prexasertib [23,35,37,38,39]. Diarrhea should be treated according to its severity, using loperamide for lower grade events, monitoring the risk of dehydration and the need for oral or intravenous fluid replacement. Criteria for dose interruption, reduction or treatment discontinuation follow the same indication reported above for emesis [25,26].

Constipation. Any-grade constipation also occurred in about one quarter of patients treated with a PARP inhibitor in phase III randomized trials, but even in this case, G3/4 events were overly rare (0–2%) (Table 1). ATR, CHK1 and WEE1 inhibitors displayed a constipation incidence of 14–38%, with no G ≥ 3 cases reported in early phase studies [23,35,37,40,42,52]. Dietary interventions and laxatives can be of help in case of constipation, while treatment interruption, dose reduction or discontinuation can be considered for more severe cases [25,26].

Other Gastrointestinal Toxicities. Dyspepsia, dysgeusia and decreased appetite were reported in 4 to 39% of patients treated with DDR-targeting agents (Table 1). Although these AEs are usually mild, they can affect patients’ quality of life as well as treatment compliance. Hence, they should be managed promptly, mainly by dietary interventions. In case of persistent dyspepsia, other causes should be ruled out and endoscopy may be sometimes indicated [25,26].

#### 2.1.3. Fatigue

Fatigue represents a class effect of DDR-targeting agents. Even though the underlying malignancy and treatment-induced anemia can partly explain this AE, its multi-factorial pathogenesis has not yet been fully elucidated [25,26]. In phase III trials, PARP inhibitor monotherapy determined fatigue in about half of the treated population, with an incidence spanning from 23% in the VELIA study to 71% in the ARIEL3 study [17,33]. However, G ≥ 3 fatigue was consistently reported in less than 10% of patients (Table 2). Among other DDR-targeting molecules, fatigue was also common and G3/4 events were registered in up to 24% of patients treated with adavosertib [23,35,37,38,39,40,42,52]. Low-intensity physical activity, psychosocial interventions and dietary modifications can all be useful in the management of this AE. Additionally, concomitant causes, such as anemia, insomnia and hypothyroidism, should be investigated and treated. Treatment discontinuation or dose reductions can be considered for higher grade or refractory fatigue [25,26,43].

#### 2.1.4. Respiratory Toxicities

Respiratory AEs of DDR-targeting agents include cough, dyspnea and rarely, pneumonitis. Evidence regarding the pathogenesis of lung toxicities is controversial. Indeed, PARP-1 inhibition seems to play a protective role towards respiratory conditions, such as interstitial fibrosis, emphysema and asthma [59,60,61]. Low grade cough and dyspnea have been registered in 11–19% of those treated with PARP inhibitors in randomized phase III trials, while data are still scarce for ATR, CHK1 and WEE inhibitor monotherapy (Table 2). In randomized controlled trials, the incidence of pneumonitis was below 1% with olaparib and niraparib, but a recent meta-analysis showed a significantly increased risk of this AE among patients treated with PARP inhibitors [25,26,62]. The same authors consulted the Food and Drug Administration Adverse Event Reporting System (FAERS) database to evaluate pneumonitis incidence in the real-world setting and reported that an increasing number of events have been described from 2015 to 2019. These AEs generally occurred within 6 months from PARP inhibitor initiation and could present with a wide range of symptoms, from dyspnea to respiratory failure [62]. According to the prescribing information, patients with new or worsening respiratory symptoms as well as radiological abnormalities should interrupt olaparib and undergo proper diagnostic work-up. In case of confirmed pneumonitis, the drug should be discontinued, and pneumonitis should be treated adequately with steroids and antibiotics [25,26].

#### 2.1.5. Neurological and Cardiovascular Toxicities

Neurological and cardiovascular disorders, including headache, insomnia, hypertension and tachycardia/palpitations, have been reported with all PARP inhibitors and also with ATR, CHK1 and WEE1 targeting agents, with a variable incidence (Table 2). According to pre-clinical evidence, activation of PARP enzymes in the central nervous system seems to have a role in neuronal death and circadian regulation, which could explain the headache and insomnia observed with PARP inhibitors [63,64], while niraparib interaction with dopamine, noradrenaline and serotine transporters as well as with the dual specificity tyrosine phosphorylation regulated kinase 1A (DYRK1A) receptor could justify the cardiovascular effects of this drug [65].

Overall, G1-2 headache was registered in phase III trials with all PARP inhibitors with a frequency varying from 10% to 33% [19,33], but the rate of G3/4 events was consistently below 3%. Low-grade headache was also reported with elimusertib and prexasertib, but not with adavosertib [23,35,37,38]. Insomnia was also reported in phase III trials with all PARP inhibitors, except talazoparib, and it was always of low grade, while it does not seem to be an AE of the other DDR-targeting agents. Despite headache and insomnia generally being mild, they can have a substantial impact on patients’ quality of life and should therefore be adequately managed with various pharmacological and non-pharmacological approaches [25,26].

Hypertension had an overall incidence of 17%, 19% and 11% in the PRIMA, NOVA and NORA trials. Grade 3/4 events were infrequent but not negligible in these studies (6%, 8% and 1%, respectively). Tachycardia and/or palpitations were also present in about 10% of patients and were always low-grade [14,44,45]. Given this data, blood pressure and heart rate monitoring are advisable during niraparib treatment to determine whether pharmacological treatment along with niraparib dose reduction are required [25,26,66].

#### 2.1.6. Secondary Malignancies

Cases of myelodysplastic syndromes/acute myeloid leukemia (MDS/AML) have been registered after exposure to PARP inhibitors among patients with ovarian cancer and other solid tumors [67]. The causal relationship between PARP inhibition and the onset of secondary hematological malignancies is not fully clear; however, induction of clonal hematopoiesis and epigenetic modifications have been proposed as possible explanations [68,69].

Even though these events are rare, with an estimated incidence of 0.5–1.4% in randomized clinical trials, a recent meta-analysis confirmed that the treatment with a PARP inhibitor significantly increases the risk of secondary hematological malignancies [67]. Additional data from pharmacovigilance registries showed an increasing number of MDS/AML from 2015 to 2020, mainly in ovarian cancer patients treated with olaparib. Cases have also been registered in subjects receiving niraparib, rucaparib or veliparib, but not talazoparib [67,70]. According to these data, the median latency to MDS/AML onset was 15–18 months, the most common sign at presentation was anemia, followed by thrombocytopenia and almost half of events display a fatal outcome [67,70]. In case of prolonged cytopenia in patients receiving a PARP inhibitor, underlying causes, such as iron or vitamin deficiency, should be promptly excluded. Whether cytopenia remains unexplained, bone marrow aspiration should be performed and if there is a diagnosis of MDS/AML, PARP inhibitor must be permanently discontinued [25,26].

#### 2.1.7. Laboratory Alterations

Elevated levels of serum creatinine have been documented in about 10–15% of patients receiving PARP inhibitors (Table 2). These drugs can interfere with several renal transporters, such as MATE1, MATE2-K, OCT1 and OCT-2, determining an increase in serum creatinine without actually affecting kidney function [71,72]. Hence, alternative ways to assess renal function (e.g., radionuclide renal scan) should be considered in case of creatinine elevation before reducing the dose of PARP inhibitor [26,71]. Increase of creatinine levels was observed in 3/25 patients treated with adavosertib in a phase I trial [40], while data about this AE are not available for elimusertib and prexasertib.

Elevation of alanine and/or aspartate aminotransferases (ALT/AST) was frequent with rucaparib in the ARIEL3 study (34% any grade, 10% G3/4), but it was mainly transient and self-limiting [17], while transaminitis was not a common toxicity in the other phase III randomized trials of PARP inhibitors (Table 2). Increased ALT/AST levels were also reported in early-phase studies of adavosertib, but not with elimusertib and prexasertib [42,52]. Management of rucaparib-related ALT/AST elevation includes treatment discontinuation for G4 events and for G3 toxicities with concomitant signs of liver disfunction (including bilirubin or alkaline phosphatase abnormalities). In these cases, rucaparib should be withheld until ALT/AST levels return to G ≤ 2. Dose reduction can be considered for G3 transaminitis, while this is mandatory in cases of G4 events [73].

### 2.2. Dose Interruptions, Reductions and Treatment Discontinuations

**Figure 2:**
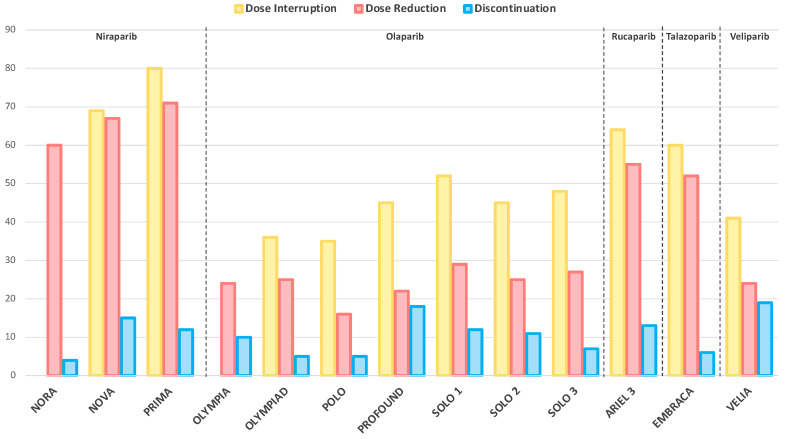
Dose interruptions, reductions and treatment discontinuations of PARP inhibitor monotherapy in phase III randomized trials. The reported dose interruptions, reductions and treatment discontinuations are due to adverse events of any cause, with the exception of NORA and NOVA trials, where they are specifically related to treatment emergent adverse events. Data about dose interruptions are not available for the NORA and OlympiA trials.

Treatment delays, dose modifications and permanent discontinuations mirror the overall toxicity burden of a therapeutic regimen. Figure 2 summarizes the incidence of these events in the experimental arms of phase III trials evaluating PARP inhibitor monotherapy.

The highest rate of dose interruptions and reductions was registered with niraparib, followed by rucaparib and talazoparib [14,17,19,45], whereas olaparib and veliparib displayed lower incidences [12,13,33]. High rates of permanent discontinuation due to toxicities were registered with veliparib in the VELIA trial (19%) and with olaparib in the PROFOUND (18%) trial, while they were less frequent with olaparib in the OlympiAD study and with niraparib in the NORA study (4% and 5%, respectively) [11,13,17,44].

Given the scarce number of patients who received an ATR, CHK1 or WEE1 inhibitor monotherapy in early phase trials, no conclusion can be drawn from the registered rates of dose interruption, reduction or discontinuation of these drugs.

## 3. Combination Regimens

**Figure 3:**
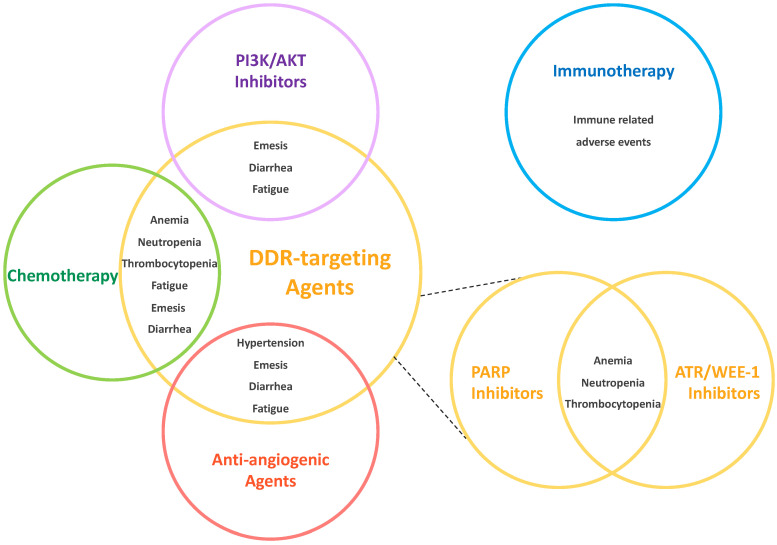
Potential overlapping toxicities of DDR-targeting agent combinations. The circles represent the different class of drugs, and the overlapping sections include the shared toxicities of these molecules, as reported in clinical trials. Immunotherapy did not show significant overlapping adverse events, therefore is represented away from DDR-targeting agents (blue circle, on the upper right). Combinations of different DDR-targeting agents (i.e., PARP inhibitors and ATR or WEE-1 inhibitors) present a significant overlap of hematological toxicities (smaller yellow circles, on the bottom right). Figure Legend: AKT: Protein Kinase B; ATR: Ataxia Telangiectasia and Rad3-Related Protein; DDR: DNA Damage Repair; PARP: Poly-ADP-Ribose-Polymerase; PI3K: Phosphatidyl Inosytol-3 Kinase; WEE1: G2 Checkpoint Kinase (WEE1).

To increase the pharmacological activity of DDR-targeting agents and circumvent mechanisms of resistance, several combinatorial strategies have been proposed and tested in clinical trials [7,74]. Although some of these approaches already showed encouraging signs of activity [7,74], they inevitably carry a higher toxicity burden, especially in cases of overlapping AEs (Figure 3). Safety results from the main trials exploring DDR-targeting agents’ combination regimens are reported below.

### 3.1. DDR-Targeting Agents and Chemotherapy

Combining DDR-targeting agents with chemotherapy has a strong biological rationale, since chemotherapy induces DNA damage while DDR-targeting agents hamper cells’ capacity in repairing it, eventually enhancing cytotoxicity [75,76]. Additionally, ATR, CHK1 or WEE1 control cell-cycle progression and their inhibition, in association with chemotherapy, induces mitotic catastrophe [21,77,78]. In the last decade, different regimens incorporating DDR-targeting agents with one or more chemotherapeutic drugs have been tested in several tumor types. As expected, overlapping toxicities, especially hematological and gastrointestinal, represented a major concern for the development of these combinations. Overall, PARP inhibitors seem to be better tolerated than ATR or WEE1 inhibitors when combined with chemotherapy, as emerges from randomized trials (Table 3).

Among PARP inhibitors, olaparib and veliparib in combination with chemotherapy, have been the most investigated. Hematological AEs represented the main toxicities in early phase non-randomized trials, with different incidence and severity according to the safety profile of the chemotherapeutic partner [90,91,92,93,94,95]. However, the addition of a PARP inhibitor to chemotherapy inconsistently increased the rate and grade of hematological and non-hematological AEs in randomized studies. In the phase III VELIA trial, ovarian cancer patients treated with carboplatin, paclitaxel and veliparib experienced higher rates of any grade and G3/4 neutropenia, anemia and thrombocytopenia compared with those receiving chemotherapy only [33]. Similar results emerged in a phase II randomized trial comparing veliparib plus carboplatin and etoposide to carboplatin and etoposide alone in small cell lung cancer patients [81]. Conversely, PARP inhibitors did not significantly modify the safety profile of standard chemotherapeutic regimens in several randomized trials across different tumor types, including breast, gastric, pancreatic and lung cancer [80,82,83,84,85].

In phase I trials testing ATR inhibitors berzosertib and ceralasertib with chemotherapy, hematological AEs (neutropenia, anemia and thrombocytopenia), fatigue and emesis were the most common toxicities [22,96,97,98]. Two randomized phase II studies confirmed that the addition of berzosertib to gemcitabine in ovarian cancer patients and to cisplatin/gemcitabine in urothelial cancer patients increased the incidence and severity of hematological toxicities and the rate of nausea and vomiting [21,87].

Hematologic and gastrointestinal toxicities represented the main AEs also in early phase trials combining the WEE1 inhibitor adavosertib with chemotherapeutic agents [99,100,101]. Consistently, in phase II randomized studies, the addition of adavosertib to gemcitabine or carboplatin/paclitaxel in ovarian cancer patients determined more G ≥ 3 hematological toxicities, especially anemia and thrombocytopenia, as well as any-grade vomiting and diarrhea [24,77].

### 3.2. DDR-Targeting Agents and Anti-Angiogenic Agents

Combinations of PARP inhibitors and anti-angiogenic agents have been tested in epithelial ovarian cancer, where these compounds proved to be effective as monotherapy [102]. Early phase trials combining these classes of drugs have been conducted even in other tumor types, such as breast and pancreatic cancer [103,104]. The simultaneous use of PARP and angiogenesis inhibitors seem to exert a synergistic effect. Indeed, anti-angiogenics-induced hypoxia might impair homologous recombination functioning, while PARP inhibition seems to affect tumor angiogenesis [102,105]. Additionally, these agents present limited overlapping toxicities as confirmed by randomized trials, further fostering their development in combination regimens (Table 3).

The randomized phase II AVANOVA trial compared niraparib plus the Vascular Endothelial Growth Factor-A (VEGF-A) inhibitor bevacizumab to niraparib alone in platinum-sensitive recurrent ovarian cancer patients [88]. Nausea, vomiting, fatigue, hypertension and anemia were the most common toxicities in both groups. However, only G1-2 emesis and hypertension (including G ≥ 3 events) occurred more frequently among individuals receiving the combination [88]. In the phase III PAOLA-1 trial, patients with advanced high grade ovarian cancer were treated with olaparib plus bevacizumab or placebo plus bevacizumab as first line maintenance therapy [16]. Anemia, emesis and fatigue were more common in the experimental arm, whereas hypertension was more frequent in the control group [16]. Overall, no signs of overlapping toxicities emerged. Olaparib has also been tested in association with the oral inhibitor of VEFG receptors 1-3 cediranib [89,103,104,106]. Updated safety results from a randomized phase II trial showed a higher rate of AEs among ovarian cancer patients treated with olaparib plus cediranib compared to olaparib alone. In this study, fatigue seems to be the only overlapping toxicity, while anemia, nausea and vomiting are mainly attributable to olaparib and hypertension and diarrhea to cediranib [107].

No data are available for combinations of anti-angiogenics and DDR-targeting agents different from PARP inhibitors.

### 3.3. DDR-Targeting Agents and Immunotherapy

Another strategy with a strong mechanistic rationale is to combine DDR-targeting agents with immune checkpoint inhibitors, since DNA-repair and immune response are intertwined biological processes [108]. A dysfunctional DNA repair machinery leads to the accumulation of genomic damages, which are recognized by the immune system and activate the immunity through the cytosolic DNA–cyclic GMP/AMP synthase complex/stimulator of the interferon genes (cGAS/STING) pathway [108,109,110]. On the other hand, DDR alterations seem to exert an immunosuppressive effect, by increasing the expression of programmed death-ligand 1 (PD-L1) in cancer cells [111]. Supported by this pre-clinical evidence, a plethora of trials have evaluated PARP inhibitors in combination with anti-programmed death 1 (PD1)/PD-L1 agents and many others are ongoing, while preliminary results are also available from early phase studies combining ATR and WEE1 inhibitors with immune-checkpoint inhibitors [7]. Given the unique toxicity spectrum of immune-therapeutic agents [112], the overlap of AEs is not expected when they are combined with DDR-targeting agents.

Niraparib and the PD-1 inhibitor pembrolizumab as well as olaparib and PD-1 inhibitor durvalumab were tested in phase I/II studies in different tumor types [113,114,115,116,117,118,119]. Most common toxicities with both combinations were nausea, anemia and fatigue, while thrombocytopenia, including G ≥ 3 events, was common with niraparib. The rate of immune-related AEs was in line with the one reported in single agent studies (15–30%) [112], with G ≥ 3 events occurring in <10% of cases [113,114,115,116,117,118,119]

Durvalumab has also been tested with the ATR inhibitor ceralasertib in a phase II trial enrolling patients with advanced melanoma. Adverse events were consistent with those expected from single agents and were mainly related to ceralasertib (anemia, thrombocytopenia, decreased appetite and nausea), with no signs of increased rate of immune-related AEs [120].

### 3.4. Other Combination Strategies

The Phosphatidyl Inosytol-3 Kinase (PI3K)/AKT pathway plays a pivotal role in several tumor types, but its inhibition has led to conflicting results thus far [121,122,123]. According to pre-clinical and translational models, targeting PI3K/AKT pathway may exert a synergistic effect toward PARP inhibition [124,125]. Despite PI3K/AKT inhibitors presenting a complex safety profile [121,122], their AEs are mainly different from those of PARP inhibitors and no cumulative toxicities are expected by associating these agents. Indeed, hyperglycemia, depression and transaminases elevation were the most concerning toxicities with olaparib and buparlisib (i.e., a non-selective PI3K inhibitor) and all of them are attributable to buparlisib [126]. The association of the alpha-subunit-specific PI3K inhibitor alpelisib and olaparib was better tolerated, even though hyperglycemia and transaminitis were reported even with this combination [127]. Olaparib has also been combined with the AKT inhibitor capivasertib, with diarrhea, emesis and asthenia as most frequent toxicities, but mainly G 1-2 [128]. Overall, nausea, vomiting, diarrhea and fatigue represent potential overlapping toxicities of PARP inhibitors and PI3K inhibitors as their high incidence in the abovementioned phase I trials suggests [126,127,128], but randomized trials are needed to elucidate this hypothesis.

Lastly, early phase trials are exploring the possibility to combine different DDR-targeting agents, such as PARP inhibitors and ATR or CHK1 or WEE1 inhibitors. This strategy is supposed to overcome PARP inhibitors’ resistance and to enhance their efficacy in patients with a proficient homologous recombination machinery [129].

Even though these drugs display similar toxicities, preliminary data suggest that they can be combined safely, adopting dose and schedule adjustments [130]. The association of olaparib and ceralasertib seems to be better tolerated compared to olaparib and prexasertib, especially in terms of hematological toxicities [131,132,133]. However, additional studies are necessary, even in this case, to clarify the safety profile of these combinations.

## 4. Future Challenges and Perspectives

A potential application of DDR-targeting agents is their combination with multiple molecules. However, as the number of combined agents increases, so does the rate of expected toxicities. Results from a phase I trial testing chemotherapy plus veliparib and bevacizumab in platinum-sensitive recurrent ovarian cancer showed a considerable incidence of AEs with dose-limiting toxicities (thrombocytopenia, neutropenia, hypertension and sepsis) in 9/12 treated patients [134]. In the same setting, chemo-free regimens, including olaparib, durvalumab and bevacizumab or niraparib, dostarlimab (i.e., a PD-1 inhibitor) and bevacizumab determined anemia, neutropenia, hypertension and fatigue, as most frequent G ≥ 3 toxicities. In these studies, 16% and 34% of patients discontinued one or more drug, respectively [135,136]. Other trials are evaluating multi-drug combinations in various diseases and settings (NCT03737643, NCT03842228, NCT04216316) but safety results are still awaited.

The dentification of biomarkers to discriminate subjects at higher risk of developing toxicities with DDR-targeting agents could guide therapeutic choices in the future. The presence of *BRCA1/2* or other germline mutations has been evaluated as a toxicity-predisposition factor. In a post-hoc analysis of the GOG9923 trial, germline *BRCA1/2* mutated patients treated with carboplatin, paclitaxel and veliparib with or without bevacizumab did not display an increased risk of AEs compared to wild-type subjects [137]. Previous evidence suggests that inherited mutations, including *BRCA1/2*, *PALB2*, *TP53* and *CHEK2*, are frequent among pre-treated cancer survivors with therapy-related MDS/AML [138,139,140]. However, the presence of a germline mutation was not consistently associated with the risk of MDS/AML in trials of DDR-targeting agents [140,141]. More recently, a correlative study of ARIEL2 and ARIEL3 trials evaluated whether the presence of pre-existing clonal hematopoiesis of indeterminate potential (CHIP) may influence the development of secondary MDS/AML. Of the 10 CHIP-related genes analyzed, *TP53* variants showed a correlation with the onset of hematological neoplasms [140]. Further studies will be necessary to elucidate the role of germline mutations, to confirm the preliminary evidence about *TP53* CHIP and to discover new biomarkers of toxicity.

## 5. Conclusions

Among DDR-targeting agents, some molecules are already available and largely used in clinical practice (i.e., PARP inhibitors) while others (e.g., ATR, CHK1 and WEE1 inhibitors) are being tested in clinical trials. PARP inhibitors display a well-known safety profile with established indications for toxicity management. Heterogeneity in the toxicity burden of these drugs likely mirrors the different pharmacokinetic features of the compounds, including their different potency in PARP inhibition and trapping and their off-target kinase effects. Indeed, talazoparib display the highest potency followed by nirabarib, whereas veliparib is the weaker inhibitor [142,143].

Data about ATR, CHK1 and WEE1 inhibitor AEs are still accumulating. Hematological and gastrointestinal AEs are expected with all these compounds, but they are usually manageable with dose adjustments and supportive measures, when needed. Particular attention should be paid to rare but severe AEs, such as pneumonitis or MSD/AML. Safety may represent a challenge when DDR-targeting agents are associated with other molecules. Indeed, combinations with chemotherapy proved to be considerably toxic in some cases, while combinations with anti-angiogenic agents as well as with immune-checkpoint inhibitors were usually better tolerated. Taking into account treatment toxicities and their potential overlap is paramount for the development of multi-drug combination regimens. Future translational research focusing on toxicity-predisposing factors could further refine treatment personalization and assist therapeutic choices. Overall, as DDR-targeting agents are expanding their role in the treatment of solid tumors, a widespread knowledge of their safety profile is mandatory among clinicians who should promptly recognize and properly manage these AEs to allow treatment continuation with the final aim of improving patient outcome and quality of life.

## Figures and Tables

**Table 1:** Main hematologic and gastrointestinal toxicities reported with PARP inhibitor monotherapy in phase III randomized trials and with other DDR-targeting agents monotherapy in early phase trials.

Class	Molecule	Trial	Ref.	Anemia		Thrombocytopenia		Neutropenia		Nausea		Vomiting		Diarrhea		Constipation		Dyspepsia		Dysgeusia		Decreased Appetite	
				Any G	G ≥ 3	Any G	G ≥ 3	Any G	G ≥ 3	Any G	G ≥ 3	Any G	G ≥ 3	Any G	G ≥ 3	Any G	G ≥ 3	Any G	G ≥ 3	Any G	G ≥ 3	Any G	G ≥ 3
**PARPi**	**NIRAPARIB**	**NORA**	[44]	53%	15%	55%	11%	59%	20%	53%	0%	32%	2%	14%	0%	30%	<1%	NR	NR	NR	NR	18%	0%
		**NOVA**	[14]	50%	25%	61%	34%	30%	20%	74%	3%	34%	2%	19%	<1%	40%	<1%	11%	0%	10%	0%	25%	<1%
		**PRIMA**	[45]	63%	31%	73%	42%	43%	20%	57%	1%	22%	<1%	19%	<1%	39%	<1%	NR	NR	NR	NR	19%	1%
	**OLAPARIB**	**OlympiA**	[46]	24%	9%	NR	NR	16%	5%	57%	1%	23%	1%	18%	<1%	NR	NR	NR	NR	12%	0%	13%	<1%
		**OlympiAD**	[47]	40%	16%	NR	NR	27%	9%	58%	0%	32%	0%	21%	<1%	13%	<1%	NR	NR	NR	NR	17%	0%
		**POLO**	[12]	27%	11%	NR	NR	NR	NR	45%	0%	20%	1%	29%	1%	23%	0%	NR	NR	NR	NR	25%	3%
		**PROfound**	[48]	50%	23%	NR	4%	NR	4%	43%	2%	20%	2%	21%	<1%	19%	0%	NR	NR	NR	NR	31%	2%
		**SOLO 1**	[49]	40%	22%	11%	1%	24%	9%	78%	1%	40%	<1%	35%	3%	28%	0%	17%	0%	22%	0%	20%	0%
		**SOLO 2**	[50]	46%	21%	17%	3%	24%	8%	76%	3%	40%	3%	34%	1%	24%	0%	15%	0%	19%	0%	23%	1%
		**SOLO 3**	[10]	51%	21%	12%	4%	23%	10%	65%	1%	38%	1%	28%	0%	12%	0%	11%	0%	NR	NR	NR	NR
	**RUCAPARIB**	**ARIEL 3**	[17]	37%	19%	28%	5%	18%	7%	75%	4%	37%	4%	32%	1%	37%	2%	15%	<1%	39%	0%	23%	1%
	**TALAZOPARIB**	**EMBRACA**	[19]	53%	39%	27%	15%	35%	21%	49%	<1%	25%	2%	22%	<1%	22%	<1%	NR	NR	NR	NR	21%	<1%
	**VELIPARIB**	**VELIA**	[33]	17%	7%	20%	7%	17%	5%	56%	5%	34%	2%	19%	<1%	12%	0%	NR	NR	NR	NR	11%	<1%
**ATRi**	**ELIMUSERTIB**	**NCT03188965**	[35]	82%	82%	45%	18%	73%	55%	50%	9%	19%	5%	23%	5%	14%	0%	NR	NR	NR	NR	14%	0%
**CHEK1i**	**PREXASERTIB**	**NCT02203513**	[23]	93%	11%	82%	25%	97%	93%	64%	0%	29%	4%	39%	7%	11%	0%	4%	0%	NR	NR	NR	0%
		**NCT02203513**	[38]	99%	33%	89%	11%	89%	89%	33%	0%	0%	0%	44%	0%	0%	0%	0%	0%	NR	NR	NR	NR
		**NCT01115790**	[37]	33%	14%	46%	16%	92%	89%	15%	0%	NR	NR	8%	0%	NR	NR	NR	NR	NR	NR	NR	NR
		**NCT02735980**	[39]	40%	15%	54%	27%	72%	66%	23%	0	13%	<1%	14%	<1%	10%	0%	NR	NR	NR	NR	25%	3%
**WEE1i**	**ADAVOSERTIB**	**NCT03668340**	[42]	68%	24%	62%	17%	44%	32%	62%	9%	41%	6%	85%	6%	38%	0%	NR	NR	21%	0%	32%	3%
		**NCT01748825**	[41]	68%	21%	45%	13%	34%	22%	81%	7%	69%	12%	65%	5%	NR	NR	NR	NR	NR	NR	NR	NR

Table Legend: G grade; NR not reported.

**Table 2:** Other selected toxicities reported with PARP inhibitor monotherapy in phase III randomized trials and with other DDR-targeting agent monotherapy in early phase trials.

Class	Molecule	Trial	Ref.	Fatigue		Increased AST/ALT		Increased Creatinine		Cough		Dyspnea		Headache		Insomnia		Hypertension		Tachycardia	
				Any G	G ≥ 3	Any G	G ≥ 3	Any G	G ≥ 3	Any G	G ≥ 3	Any G	G ≥ 3	Any G	G ≥ 3	Any G	G ≥ 3	Any G	G ≥ 3	Any G	G ≥ 3
**PARPi**	**NIRAPARIB**	**NORA**	[44]	25%	<1%	24%	1%	NR	NR	12%	0%	NR	NR	18%	<1%	29%	<1%	11%	1%	18%	<1%
		**NOVA**	[14]	59%	8%	NR	NR	NR	NR	15%	0%	19%	1%	26%	<1%	24%	<1%	19%	8%	10%	0%
		**PRIMA**	[45]	34%	2%	NR	NR	11%	<1%	15%	0%	18%	<1%	26%	<1%	25%	<1%	17%	6%	NR	NR
	**OLAPARIB**	**OLIMPIA**	[46]	40%	2%	NR	NR	NR	NR	NR	NR	NR	NR	20%	<1%	NR	NR	NR	NR	NR	NR
		**OLIMPIAD**	[47]	30%	3%	12%	2%	NR	NR	17%	0%	NR	NR	20%	1%	NR	NR	NR	NR	NR	NR
		**POLO**	[12]	60%	5%	NR	NR	NR	NR	NR	NR	NR	NR	NR	NR	NR	NR	NR	NR	NR	NR
		**PROFOUND**	[48]	42%	8%	NR	NR	NR	NR	11%	0%	11%	2%	NR	NR	NR	NR	NR	NR	NR	NR
		**SOLO 1**	[49]	64%	4%	NR	NR	NR	NR	17%	0%	15%	0%	23%	<1%	10%	0%	3%	<1%	NR	NR
		**SOLO 2**	[50]	67%	6%	NR	NR	11%	0%	19%	1%	12%	1%	26%	1%	7%	0%	4%	0%	NR	NR
		**SOLO 3**	[10]	52%	4%	NR	NR	<1%	<1%	NR	NR	NR	NR	16%	0%	NR	NR	NR	NR	NR	NR
	**RUCAPARIB**	**ARIEL 3**	[17]	71%	7%	34%	10%	15%	<1%	15%	0%	14%	0%	19%	<1%	15%	0%	9%	2%	NR	NR
	**TALAZOPARIB**	**EMBRACA**	[19]	50%	2%	NR	NR	NR	NR	NR	NR	18%	2%	33%	2%	NR	NR	NR	NR	NR	NR
	**VELIPARIB**	**VELIA**	[33]	23%	6%	NR	NR	NR	NR	NR	NR	NR	NR	10%	<1%	13%	1%	NR	NR	NR	NR
**ATRi**	**ELIMUSERTIB**	**NCT03188965**	[35]	68%	9%	NR	NR	NR	NR	14%	0%	14%	0%	23%	0%	NR	NR	NR	NR	NR	NR
**CHEK1i**	**PREXASERTIB**	**NCT02203513**	[23]	53%	7%	NR	NR	NR	NR	NR	NR	NR	NR	4%	0%	NR	NR	NR	NR	NR	NR
		**NCT02203513**	[38]	67%	0%	NR	NR	NR	NR	NR	NR	22%	0%	11%	0%	NR	NR	NR	NR	NR	NR
		**NCT01115790**	[37]	28%	2%	NR	NR	NR	NR	NR	NR	NR	NR	12%	1%	NR	NR	NR	NR	NR	NR
		**NCT02735980**	[39]	39%	8%	NR	NR	NR	NR	22%	0%	23%	8%	NR	NR	NR	NR	NR	NR	NR	NR
**WEE1i**	**ADAVOSERTIB**	**NCT03668340**	[42]	65%	24%	38%	9%	NR	NR	21%	0%	32%	0%	NR	NR	27%	0%	NR	NR	NR	NR
		**NCT01748825**	[41]	52%	7%	26%	0%	NR	NR	NR	NR	NR	NR	NR	NR	NR	NR	NR	NR	NR	NR

Table Legend: G: grade; NR: not reported.

**Table 3:** Comparison of toxicities between experimental and control arm in selected randomized trials of DDR-targeting agent combinations.

Partner Agent	DDR Class	DDR Molecule	Treatment Arms	Population	Trial Phase	AEs More Frequent with Combination		Ref.
						Any Grade (Δ ≥ 5%)	Grade ≥ 3 (Δ ≥ 5%)	
**Chemotherapy**	**PARP-i**	Olaparib	Pacli + Ola vs. Plcb + Ola	Advanced Gastric Cancer	III	Anemia; Diarrhea	Anemia; Neutropenia	[79]
		Rucaparib	CDDP + Ruca vs. CDDP	Triple Negative Brest Cancer (Adjuvant)	II	Fatigue; Nausea	Fatigue; Nausea Neutropenia; Vomiting	[80]
		Veliparib	CBDCA + VP16 + Veli vs. CBDCA + VP16	Small-Cell Lung Cancer	II	Anemia; Fatigue; Headache; Hypokalemia; Hyponatremia; Nausea; Neutropenia; Thrombocytopenia	Anemia; Febrile neutropenia; Hypokalemia; Hyponatremia; Neutropenia; Thrombocytopenia	[81]
			CDDP + Gem + Veli CDDP + Gem + Plcb	Stage III-IV Pancreatic Carcinoma *gBRCA*/*PALB2* mut	II *	Nausea	Anemia; Neutropenia; Thrombocytopenia	[82]
			mFOLFIRI + Veli vs. FOLFIRI	Advanced Pancreatic Carcinoma	II	NR	Dehydration; Diarrhea; Fatigue; Nausea; Neutropenia; Vomiting	[83]
			CBDCA + Pacli + Veli vs. CBDCA + Pacli + Plcb	Epithelial Ovarian Cancer (First Line)	III	Anemia; Constipation; Insomnia; Neutropenia; Thrombocytopenia	Anemia; Neutropenia; Thrombocytopenia	[33]
			CBDCA + Pacli + Veli vs. CBDCA + Pacli + Plcb	Triple Negative Brest Cancer (Neoadjuvant)	III	Diarrhea; Nausea; Neutropenia; Stomatitis; Thrombocytopenia; Vomiting	Anemia	[84]
			CBDCA + Pacli + Veli vs. CBDCA + Pacli + Plcb	Advanced Triple Negative Brest Cancer *gBRCA* mut	III	Anemia; Back pain; Cough; Diarrhea; Hypomagnesemia; Nausea; Peripheral edema	Thrombocytopenia	[85]
			CBDCA + Pacli + Veli vs. CBDCA + Pacli + Plcb	Advanced Squamous NSCLC	III	No differences ≥5%	No differences ≥5%	[86]
	**ATR-i**	Berzosertib	CDDP + Gem + Berzo vs. CDDP + Gem	Urothelial Cancer	II	Emesis; Fatigue; Peripheral edema	Neutropenia; Thrombocytopenia	[87]
			Gem + Berzo vs. Gem	Platinum Resistant Ovarian Cancer	II	Anemia; AST/ALT increase; Headache; Nausea; Neutropenia; Thrombocytopenia; Vomiting	Neutropenia; Thrombocytopenia	[21]
	**WEE1-i**	Adavosertib	Gem + Ada vs. Gem+ Plcb	Platinum Resistant Ovarian Cancer	II	Abdominal painAlopecia; Diarrhea; Constipation; Fatigue; Fever; Hypertension; Hypokalemia; Hypomagnesemia; Hyponatremia Insomnia; Nausea; Neutropenia; Thrombocytopenia; Vomiting	Anemia; Febrile neutropenia; Hypertension; Hypokalemia; Hypomagnesemia; Neutropenia; Rash Thrombocytopenia; Vomiting	[77]
			CBDCA + Pacli + Ada vs. CBDCA + Pacli + Plcb	Platinum Sensitive Ovarian Cancer *TP53* mut	II	Abdominal pain; Anemia Constipation; Diarrhea; Dyspnea; Myalgia; Nausea; Neutropenia Thrombocytopenia; Vomiting	Anemia, Diarrhea; Febrile Neutropenia; Neutropenia; Thrombocytopenia; Vomiting	[24]
**Anti--angiogenics**	**PARP-i**	Niraparib	Beva + Nira vs. Nira	Platinum Sensitive Ovarian Cancer	II	Anemia; Anorexia; Cough; Headache; Hypertension; Myalgia; Nausea; Proteinuria; Vomiting	Hypertension	[88]
		Olaparib	Beva + Ola vs. Beva + Plcb	Platinum Sensitive Ovarian Cancer	III	Anemia; Fatigue; Hypertension; Nausea; Thrombocytopenia; Vomiting	Anemia	[16]
			Cedi + Ola vs. Ola	Platinum Resistan Ovarian Cancer	II	Abdominal pain; Anorexia; Constipation; Diarrhea; Fatigue; Headache; Hypertension; Hypothyroidism; Mucositis; Proteinuria; Thrombocytopenia	Diarrhea; Fatigue; Hypertension	[89]

* Two-arm non-comparative trial. Table Legend: Δ differential incidence between experimental and control arm; Ada: adavosertib; Berzo: berzosertib; Beva: bevacizumab; CBDCA: carboplatin; CDDP: cisplatin; Cedi: cediranib; DDR: DNA damage response; FOLFIRI: folinic acid, 5-fluorouraicil and irinotecan; gBRCA/PALB2: germline BRCA/PALB2; Gem: gemcitabine; -i: inhibitors; Mut: mutated; Nira: niraparib; NA: not applicable; NR: not reported; NSCLC: non-small cell lung cancer; Ola: olaparib; Pacli: paclitaxel; Pclb: placebo; Ref: reference; Veli: veliparib; VP16: etoposide.
